# A Novel Baseline-Free Damage Detection Method Based on Path Scanning of Lamb Waves Using Mobile Transducers

**DOI:** 10.3390/s22062076

**Published:** 2022-03-08

**Authors:** Hongqiang Yuan, Kai Zhou, Xiuquan Li, Xiaolong Wei, Zeyu Yu, Qi Ma, Guofeng Du

**Affiliations:** 1 School of Urban Construction, Yangtze University, Jingzhou 434023, China; hqyuan@yangtzeu.edu.cn (H.Y.); zhouk@yangtzeu.edu.cn (K.Z.); 2 Department of Disaster Mitigation for Structures, Tongji University, Shanghai 200092, China; lixiuquan_tg@tongji.edu.cn; 3 School of Petroleum Engineering, Yangtze University, Wuhan 430100, China; 201873005@yangtzeu.edu.cn; 4 School of Electronics & Information, Yangtze University, Jingzhou 434023, China; 202073045@yangtzeu.edu.cn (Z.Y.); 2021730030@yangtzeu.edu.cn (Q.M.)

**Keywords:** baseline-free, angular scattering characteristic, path scanning, delay-and-sum imaging, mobile transducer

## Abstract

The baseline-free damage detection method of Lamb waves has the potential to obtain damage information efficiently in plate structures through damage scattering signals. However, the missing detection of damage occurs occasionally due to the angular scattering characteristic of Lamb waves. To solve this problem, a novel baseline-free damage detection approach based on path scanning at the detection region edges using mobile piezoelectric transducers is proposed herein. Several sensing points carrying separated damage scattering signals were picked out from the scanning paths. By removing the direct and boundary reflected signals, the damage signals were extracted and exported to a delay-and-sum imaging method to locate the damage. Two experiments with and without mobile transducers were conducted to validate the proposed method on an aluminum plate with artificially fabricated crack-like damage. The results show that the proposed baseline-free approach can locate the crack-like damage with high accuracy and efficiency and avoid potential loss of damage information. The proposed baseline-free method provides a novel and practical damage detection approach when considering the angular-dependent scattering characteristic of Lamb waves and can enhance the credibility of results in damage detection.

## 1. Introduction

Large-scale metal plates ubiquitously serve as crucial structural components in the aerospace, petroleum, mechanical and civil engineering industries [1,2,3]. To evaluate the conditions and avoid possible catastrophic failures caused by mechanical property degradation of the material, a timely inspection of flaws and damages and health monitoring, in the long run, is indispensable for the plate structures during service. Over the years, a broad range of sensing techniques, such as electro-mechanical impedance, fiber Bragg grating, ultrasonic guided wave and acoustic emission, have been widely implemented in structural health monitoring (SHM) of various structures. Meanwhile, the corresponding signal processing methods such as wavelet transform and empirical mode decomposition has also been substantially studied and applied [4]. Recently, the application of smartphones, high-resolution cameras, unmanned aerial vehicles, and other non-contact sensing technologies gained prominent growth in SHM due to their advantages of low labor cost, high applicative efficiency, and so forth [5]; in line with these advancements, a myriad of machine learning and deep learning-based algorithms, typified by support vector machine, Gaussian mixture, convolutional neural network, and long short-term memory network, have been studied and proposed to process sheer amount and various dimensions of data collected, as well as to provide intelligent solutions for conventional damage detection methods [6,7].

Among the aforementioned SHM techniques, the ultrasonic guided wave-based method received significant attention because of the ability of ultrasonic waves to travel in thin-walled structures over long distances and its high sensitivity to minor structural damages. According to the geometry and material property of the structure and the transducer selected for actuation and sensing, the form of the guided wave varies largely among Lamb wave, Rayleigh wave, shear-horizontal (SH) wave, and so on. In addition, the nature of multimode can further facilitate the application of guided waves as different wave modes are usually sensitive to different types of damage [8,9,10]. For plate-like structures, the Lamb wave which propagates through thin plates and shells, has become a quite apt and commonly adopted approach in SHM.

Generally, Lamb wave-based damage detection methods are performed by subtracting or comparing inspection data with a prerecorded baseline of the pristine structure [11,12,13]. The isolated signals are regarded as a specific signature to indicate the presence of structural damage. However, reliable baseline data are extremely difficult and impractical to obtain in realistic conditions due to varying environmental and operational conditions, such as fluctuations in temperature [14,15], variation in surface moisture [16,17], vibrations, loading conditions [18,19,20], and so forth. As a result, the baseline-dependent method can identify damages that are sometimes mistaken in the presence of these varying conditions. The formation and expansion of the damage are usually progressive and thus need continuous monitoring and signal comparison. Identifying continuous damage in systems remains a challenge for conventional reference data-based SHM approaches due to the lack of robust baseline data [21,22].

The obstructions caused by reference data triggered the exploration of baseline-free damage detection techniques based on Lamb waves. Through utilizing sensor-actuator pairs of identical lengths, detection methods based on instantaneous baseline techniques have been introduced by some researchers, in which the undamaged signals between sensor-actuator pairs are taken as the baseline data [23,24,25]. To overcome the limitations of length-fixed transducer pairs, Qiu et al. [26] improved the instantaneous baseline measurement by employing transducer pairs with varying lengths through distance compensation; thus, expanding the method’s adaptability for different types of structures. Furthermore, Zheng et al. [27] developed an integrated approach based on Lamb waves and electro-impedance in local and global damage detection on a plate, in which a mobile transducer set generated instantaneous baseline data. Another group of methods based on the reciprocity principle and time-reversal method have also been studied and applied in conjunction with other techniques. Jia et al. [28] proposed a modified elliptical localization method based on the reciprocity principle and the mode conversion to localize multiple defects in a metallic plate. He et al. [29] applied the principle of reciprocity loss and Ordering Points to Identify the Clustering Structure (OPTICS) and K-means intelligent clustering algorithms in the damage inspection of welded plate-like structures and achieved accurate defect localization. Huang et al. [30] exploited the reciprocity principle in circular and helical sensor arrays and found that the helical form performed much better than the circular form due to its asymmetry. Agrahari and Kapuria [31] proposed a refined time-reversal method to find the best reconstruction frequency and used an extended wave packet for computing the damage index. Kannusamy et al. [32] integrated a probabilistic imaging algorithm with the refined time-reversal methods and achieved higher accuracy and application range compared with conventional methods.

Recently, considerable literature has focused on baseline-free approaches with signal processing techniques, such as wavelet transform [33,34], random transform [35], Gaussian smoothing [36,37], and dictionary learning [38,39]. It is worth noting that Zhou et al. [40] proposed a method to separate multiple modes of Lamb waves by reconstructing pure mode basis signals calculated upon phase velocity and group velocity. The concealed damage scattered signals can be extracted by removing the unwanted components to identify the damage. Zhang et al. [41] furthered the application of the mode reconstruction method by combining it with a probability-based diagnostic imaging algorithm and achieved accurate damage localization and diagnostic imaging.

Among the extensive studies on Lamb wave-based defect detection, the angular scattering of incident waves when encountering holes [42], notches [43], and irregular shapes [44] was presented as a key issue by researchers to explore the influence of such phenomena. The scattering directivity patterns are non-negligible and suggested to be taken into consideration for sensor network design and defect detection in SHM. While the analyses of angular scattering patterns have been conducted by various means of simulation and experiment, few researchers have reported mitigating or eliminating the corresponding adverse effects with appropriate measures. As damage detection on plate-like structures is commonly accomplished based on sensor networks composed of a limited number of regularly distributed transducers, damage-scattered signals may be weak or absent due to deviations between the direction of the reflected wave and transducer location. Missing valuable damage information can weaken the accuracy and efficacy and even lead to failure in damage detection.

In this research, a novel baseline-free approach based on mobile piezoelectric transducers is proposed to cope with the missing detection problem of damage caused by the angular scattering characteristic of Lamb waves. With the path scanning strategy and the delay-and-sum imaging method, a crack-like defect was introduced and detected in an aluminum plate. The remaining parts of this study are organized as follows. The angular scattering characteristic of Lamb waves at the plates’ damage is described first. Then, the damage signal extraction method is introduced through path scanning based on a pure A_0_ mode signal collection, followed by the damage imaging method. Afterwards, damage detection experiments are carried out to verify the proposed damage detection approach. Finally, the experiment results are discussed and the conclusions are presented, along with the proposal of a strategy for practical inspections.

## 2. Angular Scattering Characteristic of Lamb Wave

Damage detection in large plate-like structures is usually conducted with distributed sensor networks [27,30]. According to particular demands and experimental conditions, the allocation of transducers is also diverse in a sensor array [45]. In general, the waves excited by one or more actuators propagate directly through the detection region and are scattered by various damage types. The signals are sensed by other transducers in the sensor network. The signal information can then be adopted to identify or further locate the damage.

The angular scattering pattern of Lamb waves is usually complicated for various types of defects [43,44,46]. The sensitivity for defect detection depends strongly on the incident angle of the Lamb waves, the geometry, and the possible damage location. For a Lamb wave excitation with incident angle $\phi_{inc}$, the surface displacement in a plate can be described in polar coordinates (r,ϕ) as [47]:

(1) $$u(r,\phi ,\omega )=A_{i}^{m}e^{-j\cdot k_{m}(\omega )\cdot r\cos(\phi -\phi_{inc})}$$

where ω is the angular frequency, k is the wavenumber and $k_{m}$ denotes the wavenumber associated with propagating mode m. The scattered field $u_{d}(r,\phi ,\omega )$ can be expressed as:

(2) $$u_{d}(r,\phi ,\omega )=H_{0}^{\left(1\right)}(k_{m}r)F_{m}(\phi ,\omega )+O(\frac{1}{\sqrt{k_{m}r}})$$

where $H_{0}^{\left(1\right)}$ denotes the Hankel function of the first kind and 0th order, O defines the asymptotic growth rate of the diffracted field for large value of r, $F_{m}$ is the far-field scattered field associated with mode m and can be expressed as:

(3) $$F_{m}(\phi ,\omega )=\sum_{n=0}^{\infty }{\left(-j\right)}^{n}C_{n}^{m}(\omega )e^{jn\phi }$$

**Figure 1:**
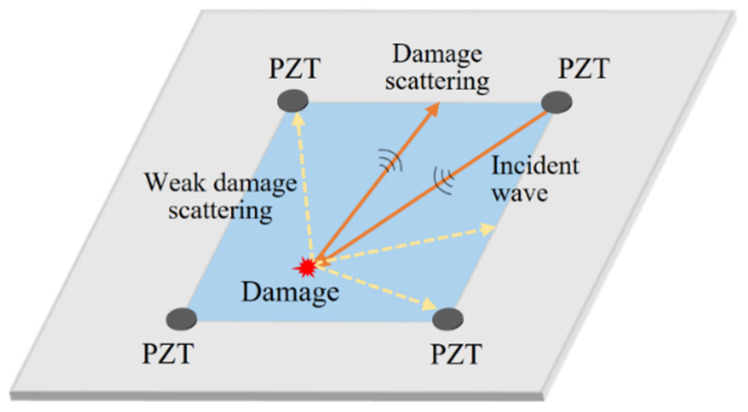
Schematic of angular scattering characteristic of Lamb waves.

As can be seen from Formulas (2) and (3), the scattered field of Lamb waves is closely related to the angle of the incident wave and the geometrical features of the damage. The angular scattering characteristic is a significant concern and needs to be considered when developing permanently attached guided-wave array systems for SHM. However, the scattered wave containing vital damage information may fail to transmit to certain sensors due to the deviation between the direction of the scattered wave and the location of the firmly bonded piezoelectric transducer (PZT), such as the wave path of the orange line in Figure 1. Meanwhile, in other directions, the scattered waves contain relatively weak or even no damage signals, as shown in the yellow dashed lines in Figure 1. As a result, the targeted damage signals may be occasionally neglected during detection in a sensor network composed of a limited number of firmly bonded PZTs.

## 3. Baseline-Free Damage Detection Method Based on Path Scanning

A baseline-free approach was introduced to deal with the possible missing detection of damage caused by the angular scattering characteristic of Lamb waves. To this end, a mobile transducer capable of sensing a relatively pure A_0_ mode was used in a specific detection scheme, termed path scanning, which can help realize the localization and imaging of damage without baseline data.

### 3.1. Pure A_0_ Mode Signal Collection

**Figure 2:**
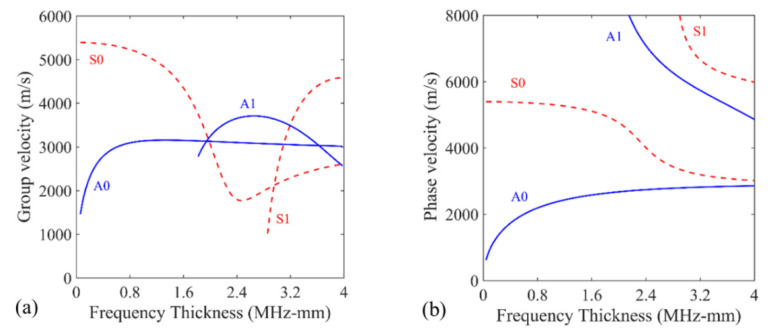
Dispersion curves of a 4 mm thick aluminum plate: (**a**) group velocity dispersion curves; (**b**) phase velocity dispersion curves.

For an isotropic plate with the material properties presented in Table 1, the group velocity dispersion curves and phase velocity dispersion curves [48] of Lamb waves are calculated as shown in Figure 2. The dispersion curves indicate that only A_0_ and S_0_ modes exist in the aluminum plate under low-frequency excitation, which will immensely reduce the signal processing complexity in damage detection.

After a long-distance propagation, the signals received by the sensor are usually complex due to the dispersive and multi-mode properties of Lamb waves. The single mode is more beneficial for detecting the reflected damage echoes as accurately as possible and using them to locate the damage. The surface-bonded PZTs using epoxy resin can sense both in-plane and out-of-plane displacements. The mixture of various wave modes usually causes many obstacles in damage signal recognition and separation.

**Figure 3:**
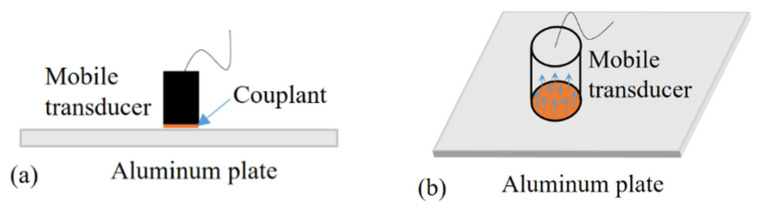
Illustration of the operating principle of the mobile transducer: (**a**) coupling of the mobile transducer and the plate; (**b**) sensing principle of the mobile transducer.

In contrast, the mobile transducer is much more sensitive to the A_0_ mode than the S_0_ mode due to the different contact patterns with the structure to be detected. As shown in Figure 3a, the mobile transducer is usually placed on the structure, with a moderate amount of couplant serving as the connecting agent to transmit the normal vibration into the structure from the transducer, or vice versa. Since the mobile transducer is not fixed and can be moved around the surface, the shear stress between the transducer and the plate is substantially reduced. Therefore, the mobile transducer is dominantly sensitive to the out-of-plane displacement and is, thus, capable of sensing a comparatively pure A_0_ mode of Lamb waves (Figure 3b). For this reason, the mobile transducer is employed in this research as a substitute for the conventional surface-bonded PZT.

### 3.2. Path Scanning Using a Mobile Transducer

**Figure 4:**
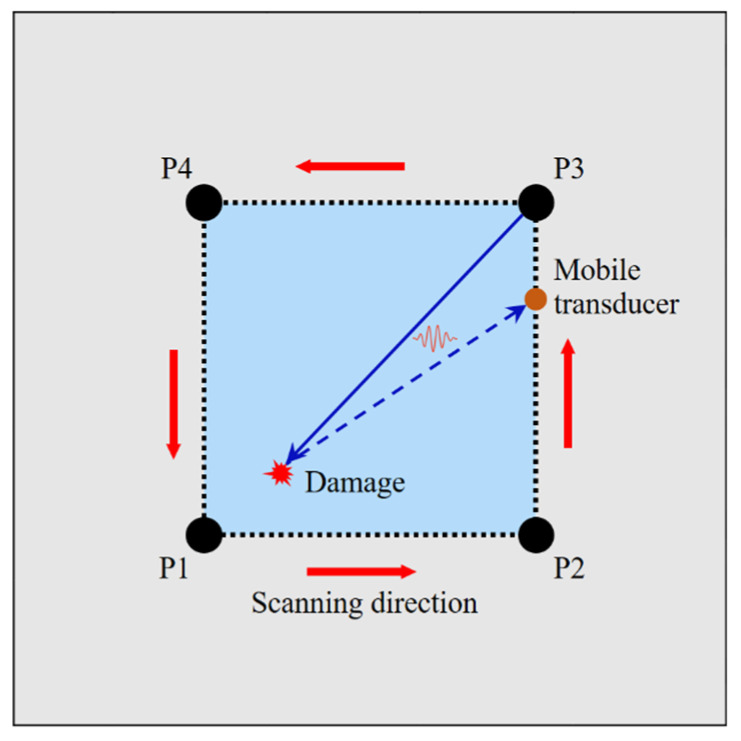
Schematic diagram of path scanning using mobile transducer.

To overcome the missing damage signals caused by the angular scattering characteristics of Lamb waves, a path scanning approach was introduced with a limited number of PZTs and a mobile transducer, as shown in Figure 4. Four PZTs (labelled P1, P2, P3, and P4) were attached to the corners of a rectangular damage detection region with epoxy resin. These PZTs served as the actuators, and a mobile transducer was employed as the sensor to receive signals along the edges of the damage detection region.

First, a modulated signal was excited on actuator P1, and the mobile transducer was exploited to move along the path P1–P2, P2–P3, P3–P4, P4–P1 successively to receive signals. Wherever the mobile transducer was placed, the received signal on the corresponding sensing point was usually composed of miscellaneous Lamb wave components, including direct waves, damage scattered waves and boundary reflected waves. Due to the change in propagation distance at different sensing points, the arrival time of different wave components varied largely, along with the shift of the mobile transducer. As a result, disparate wave components might be mixed. The recognition and extraction of the damage scattered signals were largely unavailable, apart from some specific locations. In the detection process, the mobile transducer was moved down the four paths and collected signals at corresponding locations. By comparing the received signals, sufficient sensing points with obviously separated waveforms of the damage signals could be picked out. The desired damage signal envelope could then be extracted according to the arrival time calculated from the phase and group velocities.

Then, the next actuator (P2) was excited with an identical moderated input signal, and the mobile transducer travelled along the four paths mentioned above to collect the signals. Another group of sensing points was used for the damage reflected signals. Following the same pattern, the detection scheme was subsequently applied on P3 and P4 r to expose other qualified sensing points.

### 3.3. Damage Localization and Imaging Method

After all the four rounds of path scanning were completed, the damage signal $\tilde{S}(t)$ was extracted from the signals at selected sensing points through the following formulas:

(4) $$\tilde{S}(t)=\left\{ \begin{array}{l} \begin{array}{cc} 0, & t<t_{1} \end{array}\\ \begin{array}{cc} S(t), & t_{1}\leq t\leq t_{2} \end{array}\\ \begin{array}{cc} 0, & t>t_{2} \end{array} \end{array}\right.$$

and:

(5) $$t_{1}=\frac{L_{a}}{V}+t_{s},\;t_{2}=\frac{L_{b}}{V}$$

where S(t) is the damage wave packet contained in the received signal, $L_{a}$ denotes the propagation distance of the wave directly from the actuator, $L_{b}$ denotes the entire propagation distance of the wave coming from the actuator and then being reflected from the boundary, V denotes the velocity of the selected Lamb wave mode, $t_{s}$ represents the length of the exciting wave in the time domain, and $t_{1}$ and $t_{2}$ represent the end time of the direct wave and the beginning time of the boundary-reflected wave, respectively.

**Figure 5:**
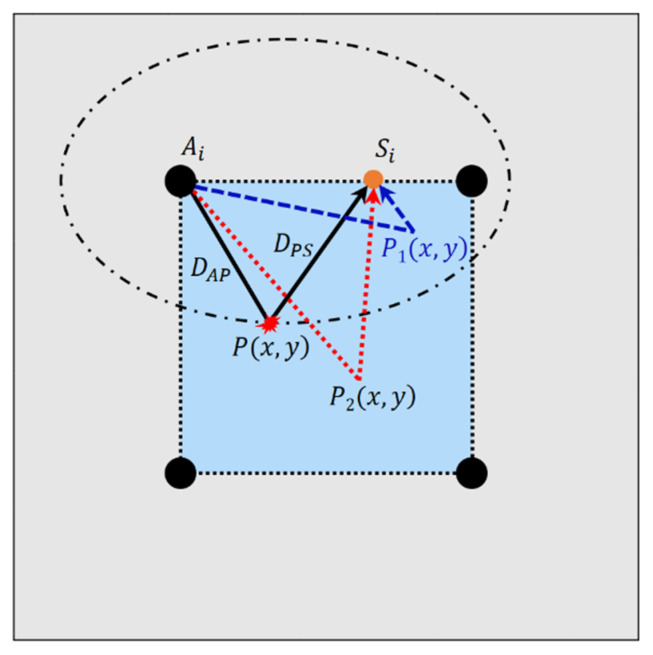
Schematic diagram of the delay-and-sum imaging method.

Subsequently, a delay-and-sum imaging method [49,50] was applied to locate the damage on the plate with the extracted signals. The imaging process was accomplished using a series of targeted sensing points on four paths and the four firmly attached PZTs. The basic schematic of the delay-and-sum imaging adopted in this research is shown in Figure 5.

Once a sensing point and corresponding actuator are selected, such as $S_{i}$ and $A_{i}$, (Figure 5), each point inside the detection area can be connected and thus form a propagation path of a Lamb wave. If damage exists at a point p(x,y), the time of flight of the damage scattered signal can be calculated as:

(6) $$t_{p}(x,y)=\frac{D_{AP}+D_{SP}}{C_{g}}$$

where $D_{AP}$ is the distance between $A_{i}$ and p(x,y), $D_{SP}$ is the distance between $S_{i}$ and p(x,y), and $C_{g}$ is the group velocity of the selected Lamb wave mode. The points connecting identical propagation paths lengths form an ellipse focused on the corresponding actuator and sensing point; hence, the trajectory of p(x,y) with a uniform time of flight is an ellipse focused on $A_{i}$ and $S_{i}$, as shown in Figure 5, while $p_{1}(x,y)$ and $p_{2}(x,y)$ are located out of the ellipse due to the discrepancy of the propagation path length.

Since the signal amplitude at the time $t_{p}(x,y)$ can be employed as the damage index (DI) to describe the probability of damage occurrence, the damage index of a certain (*i*th) path going through p(x,y) can be described as:

(7) $$DI_{i}(x,y)=H_{i}(t_{p}(x,y))$$

where H is the signal amplitude of the selected path obtained using a Hilbert transform; hence, for a certain point, there will be several paths with identical lengths and corresponding ellipses assigned with a damage index $DI_{i}(x,y)$. The average amplitude of a point in the inspection area can be expressed as:

(8) $$DI(x,y)=\frac{\sum_{i=1}^{N}DI_{i}(x,y)}{N}$$

where N is the number of paths incorporated in the calculation, and DI(x,y) is the average amplitude of that point. Through at least three disparate ellipses, the defect can be localized, and the image of the defect can be yielded with the distribution of calculated average amplitudes.

## 4. Damage Localization and Imaging Experiment

### 4.1. Experimental Setup and Procedure

**Figure 6:**
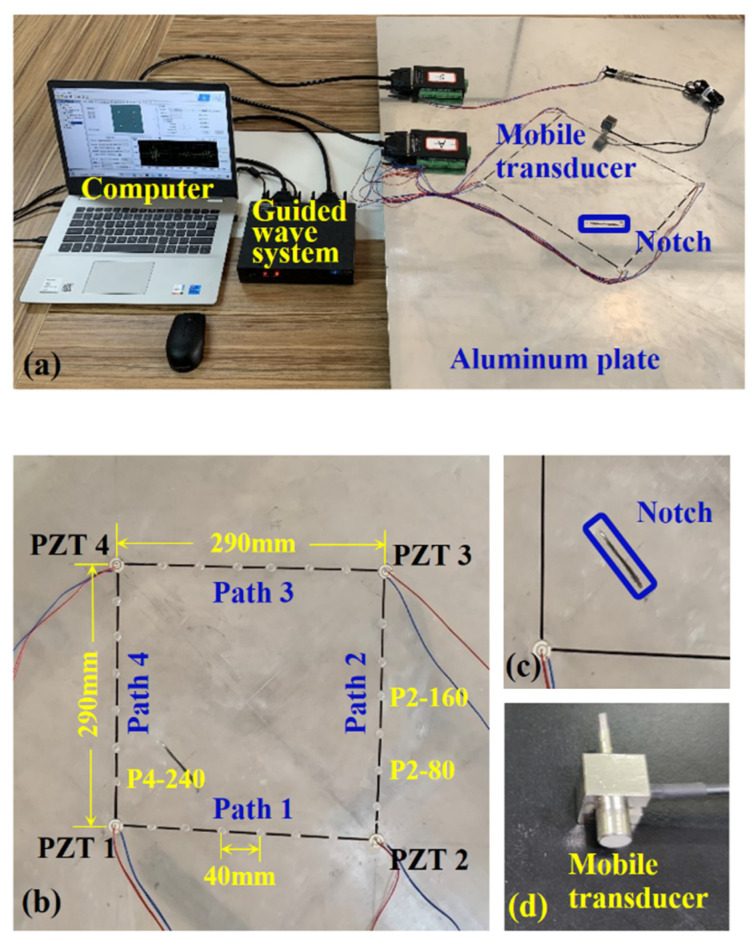
Experimental setup and relevant details: (**a**) experimental setup; (**b**) layout of the detection region; (**c**) the notch; (**d**) the mobile transducer.

A 4 mm thick aluminum plate specimen (Al 6061) of the dimensions 1200 mm × 1200 mm was employed with a squarely distributed array of four PZTs (diameter 16 mm, thickness 0.5 mm) firmly mounted on the surface, as shown in Figure 6a. Figure 6b shows the layout of the 290 mm × 290 mm detection area, and Figure 6c exhibits the through-thickness notch artificially fabricated on the specimen with a length of 40 mm and a width of 2 mm. A customized mobile transducer [21] consisting of PZT element, damping medium, shield and connection port, as shown in Figure 6d, was utilized to sense relatively pure A_0_ mode of Lamb wave propagating in the aluminum plate. A Vaseline lubricant was selected to enhance the coupling effect between the mobile transducer and the plate. An integrated guided wave-based SHM system was deployed to excite and capture guided wave signals with a 12 MHz sampling rate and 4000 sampling points.

In the experiment, a broadband 5-cycle sinusoidal tone burst waveform, with center frequencies ranging from 50 kHz to 120 kHz at a uniform increment of 10 kHz, was excited on PZT1, PZT2, PZT3, and PZT4. When each PZT was actuated, the mobile transducer was simultaneously employed to travel along the sequential route path1-path2-path3-path4 at an interval of 40 mm on each path and receive signals at corresponding sensing points. Figure 6b shows the distribution of pre-set sensing points uniformly denoted as Pm-n, where m is the serial number of the path on which the point locates and n is the distance between corresponding sensing points and the front-end PZT of that path, such as P2-80 and P4-240. After four detection rounds were completed, a group of targeted sensing points emerged with an apparently separated A_0_ mode comprised in the received signal. The corresponding damage signals were subsequently extracted and applied in defect localization and delay-and-sum imaging.

In the abovementioned scenario of damage detection based on path scanning, the mobile transducer was shifted around the paths and received signals at various locations with a uniform interval. Several specific sensing points suitable for damage localization were picked out by extracting damage information in the received signals. However, the targeted sensing points were occasionally difficult to obtain due to the absence of proper wave propagation paths due to angular scattering characteristics. To tackle this problem, the mobile transducer was moved following the travelling direction of the wave packet until a more appropriate sensing point was found.

In addition to the experiment explained above, an alternative design was also conducted with the same experiment setup free of the mobile transducer, in which the PZTs served as both actuators and sensors. The collected signals were similarly analyzed and applied in the damage localization and imaging method.

### 4.2. Results and Discussion

**Figure 7:**
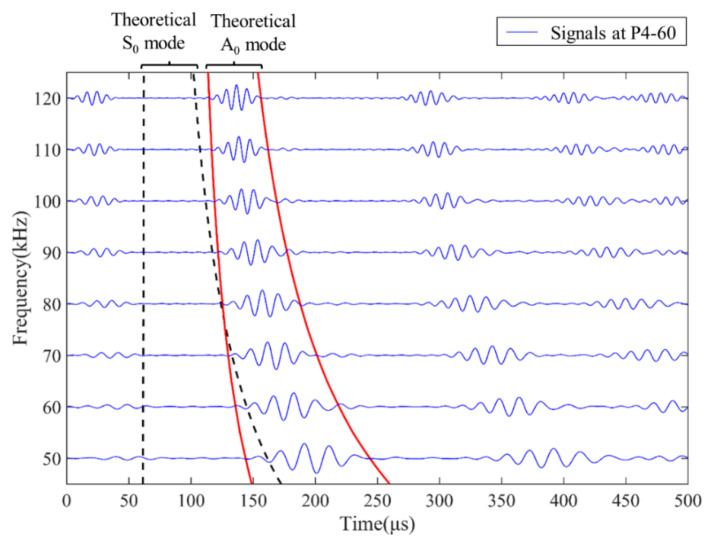
Comparison of theoretical and experimental arrival times of Lamb wave signals at different frequencies.

For the baseline-free approach introduced in this research, the mobile transducer was the key factor fulfilling the method with the specific function of sensing a relatively pure A_0_ mode. During the experiment, scattered signals were successively excited in PZT3 under 50 to 120 kHz frequencies at a fixed interval of 10 kHz, then collected by the mobile transducer at the sensing point p4-160. As shown in Figure 7, the black dashed lines and the red lines were used to frame the S_0_ mode and A_0_ mode of direct wave in the signals, respectively. The received signals excited under various frequencies showed that the A_0_ mode consistently emerged at corresponding positions in the time domain. The amplitude of each A_0_ wave-packet was apparent and large enough to be easily recognized and extracted. The S_0_ mode can scarcely be discerned from signals at corresponding positions. The comparison verified that the mobile transducer can collect relatively pure A_0_ mode and can be employed to implement the damage detection method.

**Figure 8:**
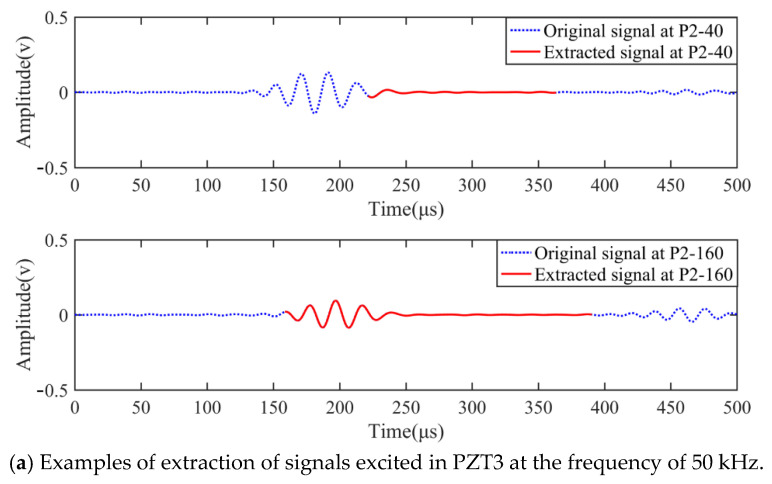

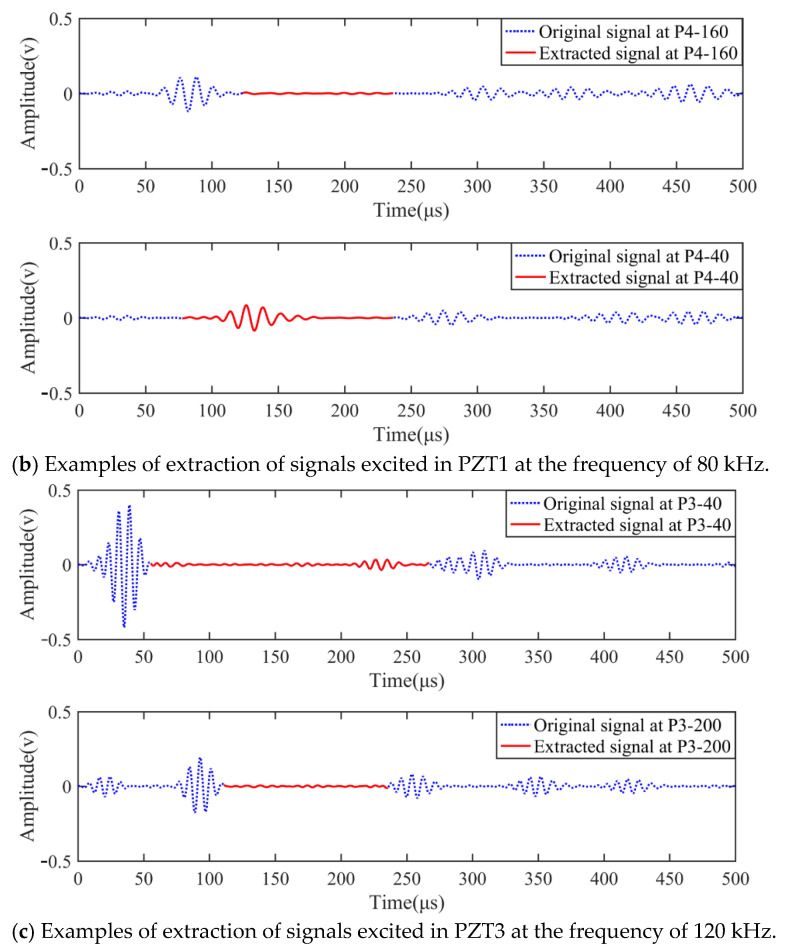
Examples of received and extracted signals at two different sensing points on the same path: (**a**) signals at P2-40 and P2-160; (**b**) signals at P4-40 and P4-160; (**c**) signals at P3-40 and P3-200.

The essence of the proposed baseline-free method is locating sensing points appropriate for damage localization and extracting damage reflected signals through the approach described in Formulas (4) and (5). Figure 8a–c exhibits the examples of original and extracted signals at two sensing points on paths 2, 3, and 4. The detection signals adopted in Figure 8a–c were excited on PZT3, PZT1, and PZT3 at 50, 8, and 120 kHz, respectively. The theoretical damage signal could be separated from the original signal on each sensing point through path scanning and signal extraction. For some sensing points, the extracted signal contained explicit damage of the wave package, such as P2-160, P4-40 and P3-40, and could be exported to the delay-and-sum imaging procedure to yield the image of damage on the detection area. Whereas, some other sensing points lacked evident damage information in the extracted signal due to improper wave propagation paths, as shown in the extracted signal at P2-160, P4-40, and P3-40. Furthermore, the wavelength of the damage signal increased along with increasing excitation frequency of the detection signal, as can be seen in the extracted signals in the three figures.

**Figure 9:**
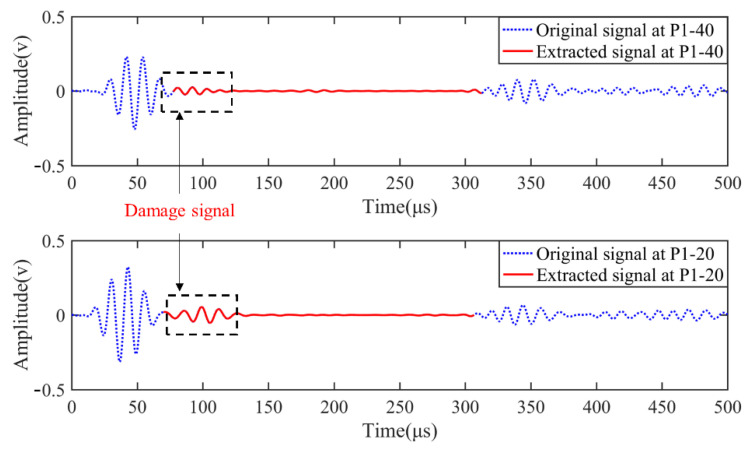
Comparison of extracted signals at P1-40 and P1-20 excited under identical conditions. The images of the damage generated with selected sensing points and corresponding actuators through the delay-and-sum imaging method are listed below.

Figure 9 compares signals received at two adjacent locations on the same path, which were both excited under the frequency of 80 kHz and transmitted from PZT1. It can be seen that the anticipated A_0_ mode (framed in black dash-line box) in the extracted signal of the initially designed sensing point P1-40 partially overlapped with other wave components. Meanwhile, the A_0_ wave packet completely separated when the mobile transducer was moved 20 mm to sensing point P1-20. Although some signals collected at different sensing points were incapable of providing useful damage information, there were various eligible ones found through path scanning and slight movement in the vicinity to reveal and locate the damage without reference data of the intact structure.

**Figure 10:**
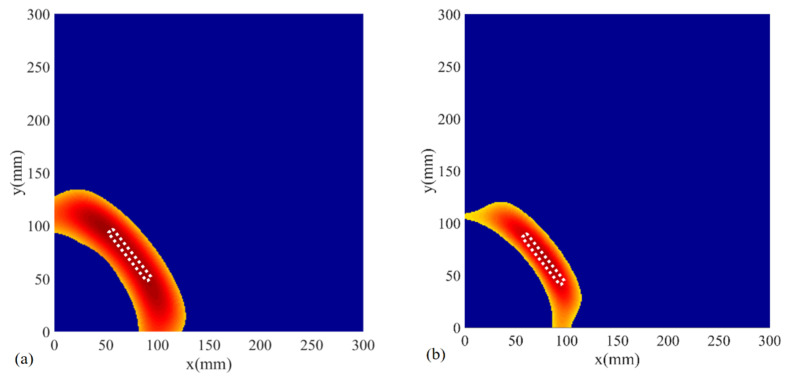

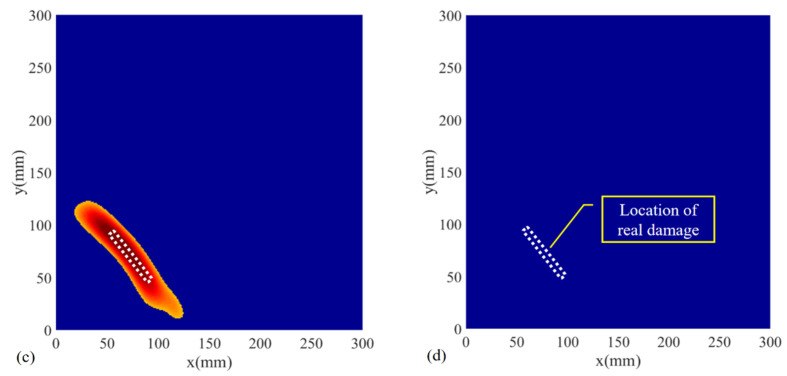
Damage imaging under various frequencies and cases: (**a**) 50 kHz using mobile transducer; (**b**) 80 kHz using mobile transducer; (**c**) 120 kHz using mobile transducer; (**d**) 50 kHz without using a mobile transducer.

The involved signals were excited at the frequencies of 50, 80, and 120 kHz (Figure 10a–c). Figure 10d shows the imaging result with signals excited at 50 kHz and received in the surface-bonded PZTs without utilizing a mobile transducer. As shown in Figure 10a–c, the defect was located and imaged accurately compared with the real notch on the aluminum plate, apart from the discrepancy of defect size induced by the wavelength imparity. Since the PZTs were firmly attached on the surface at a fixed location, scattered signals containing damage information were inevitably missed due to the lack of proper wave propagation path, thus, leading to the absence of defect image in the detection region (Figure 10d).

**Figure 11:**
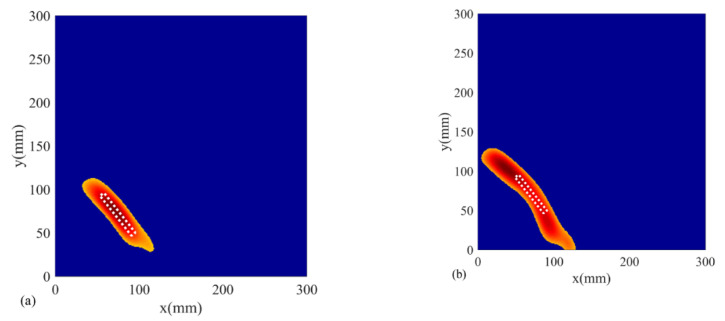
The damage image generated with a changed group velocity of Lamb wave A_0_ mode at 120 kHz: (**a**) the image generated with a group velocity of 2.8 m/ms; (**b**) the image generated with a group velocity of 3.0 m/ms.

Due to the influence of operational and environmental conditions, such as temperature, pressure, and loading, some of the acquired signals inevitably contain measurement errors in SHM. When implementing the method introduced herein, there are usually a number of appropriate sensing points on various scanning paths selected through damage signal extraction. When adopted in the delay-and-sum imaging, these selected sensing points will form a bunch of different ellipses and locate the damage with the superimposed signal amplitude; thus, is capable of reducing the impact of measurement errors to a limited extent. One of the evident consequences of measurement errors is the change in propagation velocity of the Lamb wave in the structure. As can be seen from Figure 2, the approximate group velocity of the Lamb wave A_0_ mode in the aluminum specimen is 2.9 m/ms at the frequency of 120 kHz. To verify the influence of the propagation velocity on the damage detection, two cases were studied with the group velocity of A_0_ mode changed to 2.8 m/ms and 3.0 m/ms at 120 kHz in some collected signals, respectively. The imaging results are shown below in Figure 11. In comparison with Figure 10c, the images of the damage in the two testing cases reveal the existence of damage and show a limited loss in localization accuracy. Based upon the sensing points on multi-paths, the proposed baseline-free method maintains the effectiveness and robustness of damage detection with the influence of measurement errors.

Furthermore, with the capability of detecting damage free of baseline data, the proposed method is likely to provide a solution for identifying continuous damage in various systems. Once implementing the scheme, the existence of damage can be revealed directly without comparing the signal with prior information, thus achieving the detection of continuous damage at any stage of its progress. However, the effectiveness of the proposed method at the initiation stage of damage remains to be further studied as the frequencies adopted are usually relatively low and may be insensitive to tiny structural defects.

## 5. Strategy for Practical Inspections

**Figure 12:**
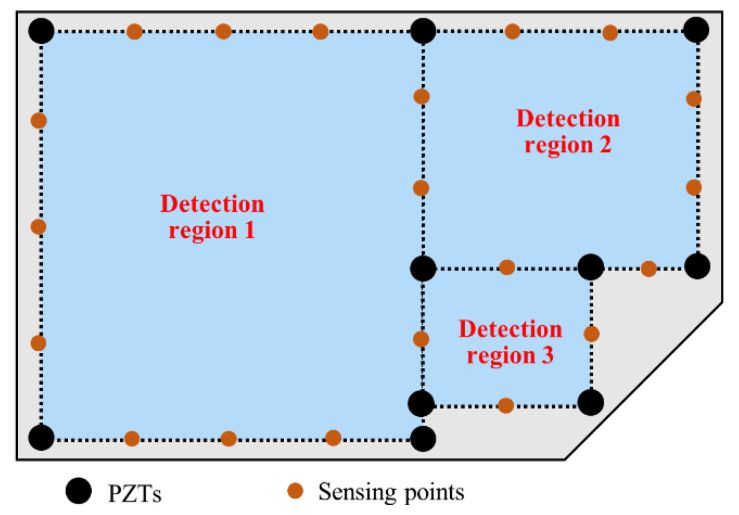
The proposed arrangement of detection regions in practical inspection of a large plate.

Finally, a strategy for practical inspections was proposed to apply the baseline-free method in various structures. As shown in Figure 12, with an appropriate distribution of PZTs, a group of detection regions varying in sizes were planned on a large plate with a specific geometry. The path scanning can then be implemented in the three detection regions, and through the signal processing and imaging the damages over the entire plate can be revealed. In practical inspections, the size of the detection region is adjustable as needed and the whole structure can be covered with the composition of a series of detection regions. The strategy makes it highly flexible and practical for the detection of structures with the proposed method.

## 6. Conclusions

A baseline-free damage detection method through path scanning and a corresponding strategy for practical inspections were proposed to solve the missing detection problem of damage caused by the angular scattering characteristics of Lamb waves. Specifically, the edges of damage detection regions were first scanned using mobile piezoelectric transducers. Then, sufficient sensing points containing separated A_0_ mode signals were selected to locate the damage using the delay-and-sum imaging method. To verify the method’s reliability, two experiments were conducted, and a series of results were obtained. Based on the experiment results, some valuable conclusions were drawn as below:

1. The path scanning-based damage detection method introduced herein can locate the crack in a plate without baseline data and maintains effectiveness under the influence of measurement errors;
2. A mobile transducer is effective and reliable in collecting pure A_0_ mode, which is beneficial for judging the damage location with high accuracy;
3. A phenomenon where the damage-scattered signals are mixed with other wave components or are weak in certain directions due to the angular scattering characteristic of Lamb waves is verified by the proposed path scanning strategy;
4. In comparison with the conventional method without using mobile transducers, the path scanning strategy can accurately obtain the necessary damage information to markedly improve the credibility of damage detection results;
5. A strategy for practical inspections is proposed to provide solutions for applying the baseline-free method in damage detection of plate structures with various geometry.

## Figures and Tables

**Table 1:** Material properties of an aluminum plate specimen.

Young’s Modulus	Poisson Ratio	Density	Thickness
70 GPa	0.33	2700 kg/m^3^	4 mm

## Data Availability

Not applicable.
